# Rat Strain Ontology: structured controlled vocabulary designed to facilitate access to strain data at RGD

**DOI:** 10.1186/2041-1480-4-36

**Published:** 2013-11-22

**Authors:** Rajni Nigam, Diane H Munzenmaier, Elizabeth A Worthey, Melinda R Dwinell, Mary Shimoyama, Howard J Jacob

**Affiliations:** 1 Human and Molecular Genetics Center, Medical College of Wisconsin, 8701 Watertown Plank Rd, Milwaukee 53226-3548, WI, USA; 2 Department of Physiology, Medical College of Wisconsin, 8701 Watertown Plank Rd, Milwaukee 53226-3548, WI, USA; 3 Department of Pediatrics, Medical College of Wisconsin, 8701 Watertown Plank Rd, Milwaukee 53226-3548, WI, USA; 4 Department of Surgery, Medical College of Wisconsin, 8701 Watertown Plank Rd, Milwaukee 53226-3548, WI, USA

**Keywords:** Rat strains, Phylogeny, RGD, Rat genome database

## Abstract

**Background:**

The Rat Genome Database (RGD) ( http://rgd.mcw.edu/) is the premier site for comprehensive data on the different strains of the laboratory rat (*Rattus norvegicus*). The strain data are collected from various publications, direct submissions from individual researchers, and rat providers worldwide. Rat strain, substrain designation and nomenclature follow the Guidelines for Nomenclature of Mouse and Rat Strains, instituted by the International Committee on Standardized Genetic Nomenclature for Mice. While symbols and names aid in identifying strains correctly, the flat nature of this information prohibits easy search and retrieval, as well as other data mining functions. In order to improve these functionalities, particularly in ontology-based tools, the Rat Strain Ontology (RS) was developed.

**Results:**

The Rat Strain Ontology (RS) reflects the breeding history, parental background, and genetic manipulation of rat strains. This controlled vocabulary organizes strains by type: inbred, outbred, chromosome altered, congenic, mutant and so on. In addition, under the chromosome altered category, strains are organized by chromosome, and further by type of manipulations, such as mutant or congenic. This allows users to easily retrieve strains of interest with modifications in specific genomic regions. The ontology was developed using the Open Biological and Biomedical Ontology (OBO) file format, and is organized on the Directed Acyclic Graph (DAG) structure. Rat Strain Ontology IDs are included as part of the strain report (RS: ######).

**Conclusions:**

As rat researchers are often unaware of the number of substrains or altered strains within a breeding line, this vocabulary now provides an easy way to retrieve all substrains and accompanying information. Its usefulness is particularly evident in tools such as the PhenoMiner at RGD, where users can now easily retrieve phenotype measurement data for related strains, strains with similar backgrounds or those with similar introgressed regions. This controlled vocabulary also allows better retrieval and filtering for QTLs and in genomic tools such as the GViewer.

The Rat Strain Ontology has been incorporated into the RGD Ontology Browser ( http://rgd.mcw.edu/rgdweb/ontology/view.html?acc_id=RS:0000457#s) and is available through the National Center for Biomedical Ontology ( http://bioportal.bioontology.org/ontologies/1150) or the RGD ftp site ( ftp://rgd.mcw.edu/pub/ontology/rat_strain/).

## Background

The use of the rat for genetics studies in Europe can be traced back to the first half of the eighteenth century. Experimentally, Crampe et al. mated an albino female to a wild gray male in 1880. In the F_1_ offspring, three mutant genes were phenotypically observed: c (albino), a (non-agouti), and h (hooded) [1]. An early effort to track new strains and substrains, focused on when rats were transferred from one lab to another, resulting in new substrains that could affect animals both phenotypically and genotypically by the resultant changes in environment, dietary conditions or breeding strategy, as well as spontaneous genetic variations. The list of codes used to designate laboratories developing and maintaining rat colonies was first published in 1973 [2]. Efforts have also been made to capture differences in phenotype by integrating microsatellite markers into the genetic linkage maps [3] and radiation hybrid maps [4]. In order to make significant comparisons, determine relationships amongst strains, and select an appropriate model for biomedical studies, knowledge of the different rat strains and their breeding approaches is crucial. The first attempt to create a phylogenetic tree for 13 inbred strains (homozygous strain produced by brother-sister mating for at least 20+ generations) using genetic markers was done by Canzian et al. [5]. This was followed by an enhanced version comprising 63 inbred strains and 214 substrains (genetically diverse inbred strains due to separation after 20 generations or separated due to any genetic difference), which was plotted using the percentage of genotypic differences [6]. Thomas et al. presented phylogenetic relationships of 48 inbred strains, using the allele size of each strain at each microsatellite locus [7]. A phylogenetic tree is also available at The National BioResource Project for the Rat in Japan [8], for 132 rat strains. Maximum parsimony analysis was used to calculate this tree ( http://www.anim.med.kyoto-u.ac.jp/nbr/phylo.aspx). Leveraging these efforts to represent relationships amongst strains, the Rat Genome Database (RGD) has created standardized data formats for capturing strain background and breeding variations to represent all registered strains in a format that is hierarchical and computable.

### RGD: a unique resource for registering rat strains

RGD is a universally accessible database that has an exclusive collection of rat genetic and genomic data curated from current research publications and direct data submission by rat researchers and rat providers. RGD currently has a catalogue of more than 2900 strains and substrains. RGD provides official assignment of rat strain symbols and names, and encourages researchers to submit strain data prior to publication through an online strain registration form ( http://www.rgd.mcw.edu/tu/strains/#StrainRegistration), to ensure proper identification of their strains in their manuscripts. RGD validates the nomenclature of the submitted strains following the nomenclature guidelines laid out by the International Committee on Standardized Genetic Nomenclature for Mouse and the Rat Genome and Nomenclature Committee [9,10]. The registered symbol and name of the strain along with a unique identifier, the RGD ID, are assigned and sent to submitters for reference in their publication. Strains from major rat resources such as the PhysGen Program for Genomic Applications (PhysGen, http://pga.mcw.edu/), Rat Resource and Research Center (RRRC, http://www.rrrc.us/), National BioResource Project (NBRP, http://www.anim.med.kyoto-u.ac.jp/nbr/Default.aspx) in Japan, and commercial rat providers such as Charles River (CRL, http://www.criver.com), Harlan Laboratories ( http://www.harlan.com/), Sigma Advanced Genetic Engineering Labs (SAGE, http://www.sageresearchmodels.com) and Transposagen ( http://www.transposagenbio.com) regularly submit strains to RGD for nomenclature and ID assignment and the creation of strain reports. These distributors mention the specific nomenclature on their websites, reminding researchers to use the correct nomenclature in their publications so that the information can be extracted and attached to the appropriate strain.

## Results and discussion

### Rat Strain Ontology

**Figure 1:**
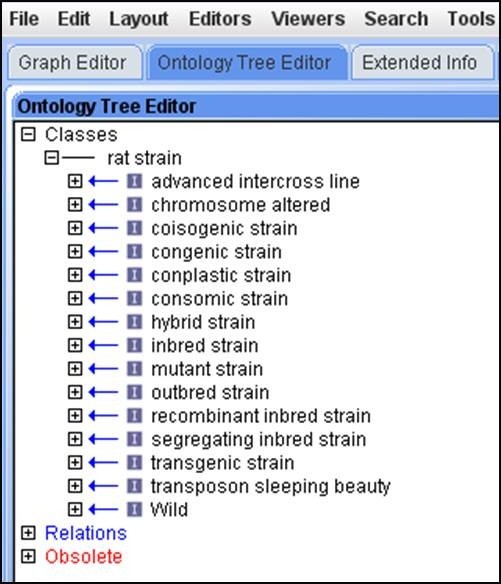
RS Ontology as viewed in OBO-Edit2 with rat strain types displayed as different first level nodes.

The Rat Strain Ontology (RS) is able to preserve the genetic background and the breeding history of strains based on their strain types. In order to do this, the strain nomenclature had to be parsed so that the parental/ancestral strains could be determined and progeny placed accordingly. In the latest version (version 5.1) of the RS Ontology, dated September 2013, there are 3503 terms, which include 13 different types of first level nodes depicting the strain types (Figure 1). 62% of the terms have one parent, including parental strains and placeholder terms used to organize the strains properly. For example, all congenic strains (strains in which a chromosomal segment has been transferred from a donor strain to a recipient strain but which is otherwise identical to the original inbred recipient strain) that are derived from the same parental strains DA and F344 are placed under the congenic placeholder DA.F344 (RS:0000237). This is further branched into congenic placeholders DA/BklArbN.F344/NHsd (RS:0001224) and DA/BklArbNsi.F344/NHsd (RS:0003211). These second level terms house the actual congenic strains, for example DA.F344-(*D10Arb21-D10Arb22*)/Arb (RS:0000210), DA.F344-(*D10Rat37-D10Arb22*)/Arb (RS:0000209). As a result, 1% of the terms have more than 3 parents. The addition of congenic substrains (substrains derived from the parental congenic strains) and mutant strains generated another layer of complexity in the vocabulary and also justify the fact that breeding techniques are crucial in defining a rat strain. Hence, it was clear that the strain background impacts the newly developed strain and its phenotype. Accordingly, these details need to be captured and represented in a format that can be used.

### Organization of the ontology

**Figure 2:**
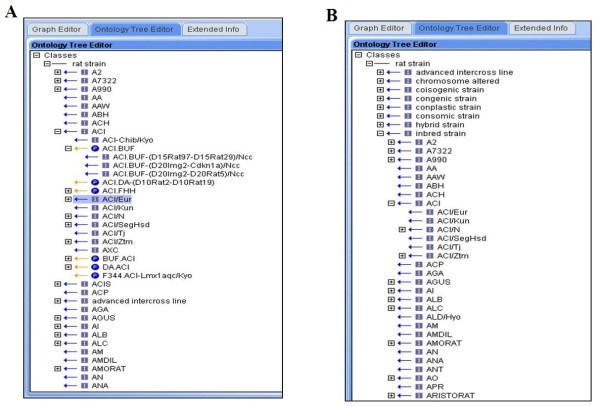
**A. Version 1.0 showing substrains and congenics in the same level node. B.** Version 4.9 showing the different strain types as first level nodes.

**Figure 3:**
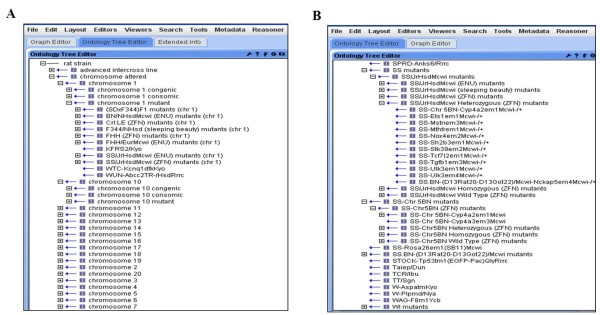
Classical tree view showing A. chromosome altered B. ZFN mutant strains.

The earlier version of this ontology (version 1.0, dated January 2009) had 2350 terms, with 76% of the terms having one parent and only three terms having more than three parents. In this format all the inbred strains and the different strains types were the main nodes. This format was not able to differentiate between the substrains and congenic strains of the same parental strain. For example, the substrain: ACI/Eur (RS:0000021) and congenic placeholder: BUF.ACI (RS:0000432) were under the parental node ACI which was a first level node under rat strains (Figure 2A). This format was replaced in the latest version 5.1 where the strain types are first nodes (Figure 2B). This major version change referred to a global change in the structure of the ontology. Therefore, ACI is an inbred strain which is placed under "inbred" with all substrains under it, whereas the related congenic strains are under the node "congenic strain". Another node recently added is the "chromosome altered" (Figure 3A). This node has all the chromosomes which can be further divided into the relevant strain types, for example: congenic, consomic (strains in which a whole chromosome has been transferred from a donor strain to a recipient strain but is otherwise identical to the original inbred recipient strain) and mutant (strains in which a gene has been modified or spontaneous mutant with an altered phenotype). All the strains in which the chromosomes have been manipulated are placed under the "chromosome altered" node, as well as under their specific categories. This helps in visualizing strains in which the same chromosome has been introgressed into different background strains making it easier to predict the genotype to phenotype associations and compare the sequence variations amongst strains.

Since techniques used to alter the chromosomes also play a crucial role in determining the strains, mutant strains are further divided. For example, mutants created by N-ethyl-N-nitrosourea (ENU) [11,12], zinc-finger nucleases (ZFN) [13] and transcription activator-like effector nuclease (TALEN) have separate nodes under the parental strains. The technique used to create the specific mutant is mentioned in parenthesis. The mutant strain in which a particular gene is mutated is placed under the relative chromosome number; for example, gene Tgfb1 (transforming growth factor, beta 1) maps to chromosome 1 in the rat, so the heterozygous mutant strain SS-Tgfb1^em3Mcwi-/+^ (RS:0003129) is placed under SS/JrHsdMcwi Heterozygous (ZFN) mutants (Figure 3B). This strain, having a mutation in chromosome 1, is also under SS/JrHsdMcwi (ZFN) mutants (chr 1) a sub-branch of "chromosome 1 mutant" under "chromosome altered". These substantial improvements have helped in making this vocabulary more robust and usable. In addition, users can now view all the homozygous, heterozygous and wild type strains under a single node.

**Figure 4:**
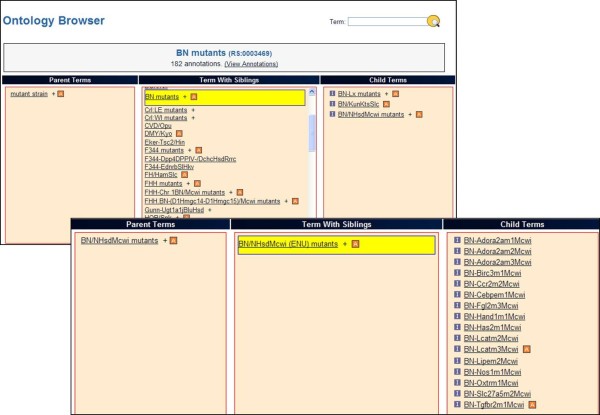
RGD Ontology Browser displaying ENU mutants.

**Figure 5:**
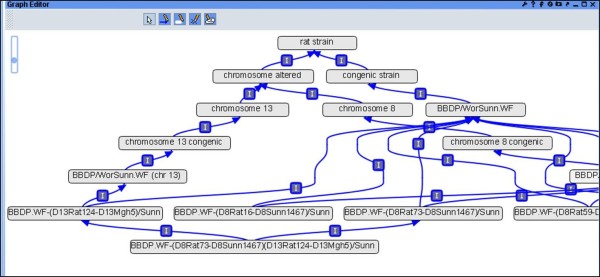
Graphic view of a congenic substrain.

**Figure 6:**
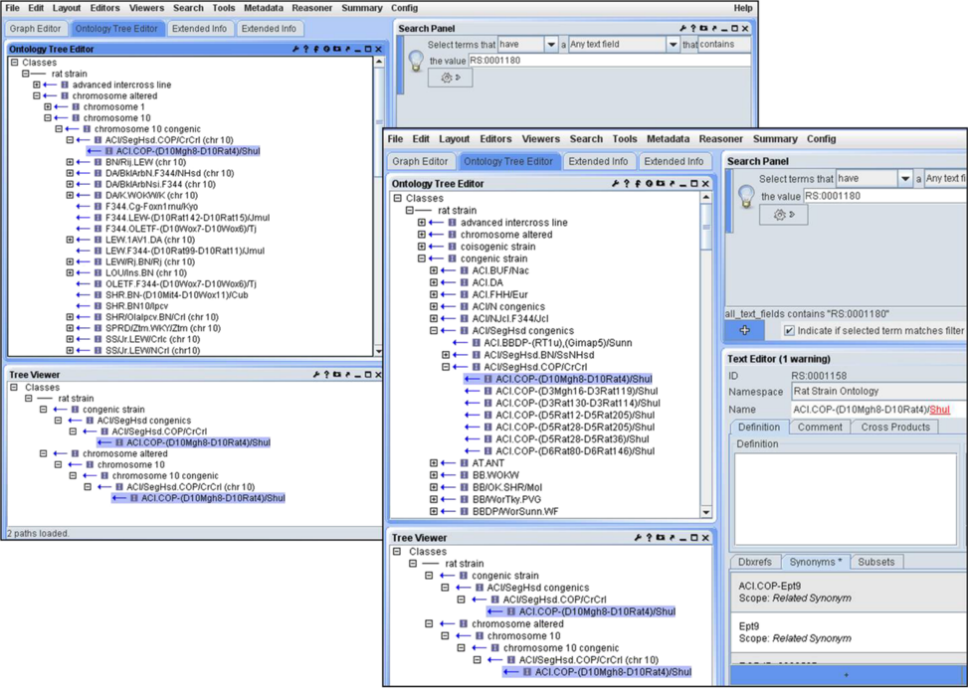
Classical tree view of the Rat Strain Ontology in OBO-Edit2.

In OBO-Edit and the RGD Ontology Browser (Figure 4), the RS Ontology can be viewed in the tree view, which can be expanded to show the child relationships [14]. Since a term has many relationships to other terms, it appears several times in the graphic view (Figure 5) or a tree view (Figure 6).

### Searching a strain in the ontology

The RGD Ontology Browser ( http://rgd.mcw.edu/rgdweb/ontology/search.html) can easily be used to search a strain. When a desired strain symbol; for example, BN is searched in the browser the result page displays the number of terms that match the searched term in all the different ontologies. Clicking on the ontology name, "RS: Rat Strains" or on the number of terms displays all the strains that have the searched term "BN" in them in the Rat Strain Ontology. By first clicking on the tree sign adjacent to "BN mutants", then BN/NHsdMcwi mutants and finally BN/NHsdMcwi (ENU) mutants, a list of all the strains is generated by this technique using the parental BN strain. A click on any individual strain takes the user to the ontology report page, where the term is displayed in a "driller" format [15], with the searched term in the middle column along with siblings, parents in the left column and children in the right (Figure 4). Clicking the "View Strain Report" option takes the user to the respective RGD strain report page which displays the RS ID. This option is available for the curated strains and not for placeholders. If a strain is searched using the general keyword search in RGD, then the result page lists all the strains. A click on the strain symbol goes to the strain report page which has the RS ID of the strain mentioned as ontology ID. Using the same example, if BN/NHsdMcwi is searched in keyword search of RGD, then the report page shows all the strains that have the searched term BN/NHsdMcwi in them. A click on the strain symbol takes the user to the individual strain report page that has the RS: 0000145, this ID links to the RGD Ontology Browser showing the different substrains derived from it. The tree view of the Rat Strain Ontology at NCBO BioPortal [16,17] also displays the hierarchy of the strains in a similar fashion.

### Expanded usage

As RGD has a vast collection of strains, selecting an appropriate strain from a list of over 2900 strains is not easy; it is here that the RS Ontology has a vital role. Users can scroll down the lists of different types of strains, or restrict their choices by "chromosome altered" and then by the different strain types. RGD’s robust usage of the RS Ontology for classifying strains makes it valuable for biologists using rats in their research, as it helps them in predicting the genomic contents a particular strain may have inherited from the parental strains. The RS Ontology is included in the RGD Ontology Browser, and the strain report pages, which have comprehensive descriptions of characteristics, origin, disease, phenotype and physiological information, behavior, drug reactions and reproductive notes make the RS Ontology annotations an important navigational tool. The rat strains that are curated from published articles are annotated by Mammalian Phenotype Ontology [18] and MEDIC disease ontology [19] which are used to conduct effective searches for strains based on disease and phenotypes. These annotations help in assigning strains to their respective disease portals.

RGD tools, such as PhenoMiner, display experimental records associated with phenotypic measurements of rat strains used in experiments. PhenoMiner has 18580 records with quantified phenotype values attached to consomic strains, 11524 values attached to inbred strains, 2870 to congenic strains, 2204 to all mutant (ZFN) strains and 2063 to all mutant (ENU) strains as of September 2013 [20]. These are entered into PhenoMiner by using the RS Ontology and three other ontologies, namely, clinical measurement (CMO), measurement method (MMO), and experimental condition ontologies (XCO) [21]. All rat QTLs are annotated to the RS Ontology to facilitate querying, retrieval and filtering of QTL data [22]. All the congenic [23] and consomic [24], strains that have an introgressed segment can be visualized in GViewer which can be accessed from the disease portals. QTL report pages have a link that leads to a narrower region which can be visualized by zooming in with GBrowse [25,26] which displays the congenic and congenic substrains that have the desired region. As stated earlier, this information is captured in the chromosome altered node of the RS Ontology.

## Conclusions

The Rat Strain Ontology is a new tool for annotating rat strains in a standardized manner which reflects the breeding history and genetic makeup of the strains to facilitate querying and retrieval, analysis and comparisons amongst strains. The latest version of the Rat Strain Ontology has been revised to classify all of the wild type, heterozygous, and homozygous strains, with the mutants further grouped under these strain subtypes. As the development process continues, new strains are continually being added and application of this vocabulary is continually expanding to allow investigators to integrate, consolidate and compare phenotypic measurement data from diverse sources.

## Methods

### Development of the ontology

This ontology is developed using OBO-Edit [27,28], a Java based tool that uses a graph-oriented approach to display and edit the ontologies. RGD currently uses OBO-Edit2 for editing and adding new strain information. In some instances, strain symbols are used as placeholders for the graph nodes in order to maintain the relationship and hierarchical structure of the ontology. For example no details are known for the parent strain ACI (RS:0000012), whereas details are known about the substrains ACI/N, ACI/Kun, ACI/SegHsd etc. So, in these cases, the parent term ACI was used as a placeholder so that the children terms could be added. Textual synonyms including the RGD ID are entered via Term Editor.

### Availability

This ontology is free and available to all users. This can be viewed in the RGD Ontology Browser at http://rgd.mcw.edu/rgdweb/ontology/search.html, as well as at the National Center for Biomedical Ontology (NCBO) BioPortal website http://bioportal.bioontology.org/ontologies/1150. Systematic versions can be downloaded from the RGD ftp site ftp://rgd.mcw.edu/pub/ontology/rat_strain/.

## Competing interests

No financial or commercial conflict of interest is declared by the authors.

## Authors’ contributions

RN prepared the manuscript, developed the ontology and curated all the rat strains. RN and MS designed the ontology. DHM, EAW, MRD, MS, HJJ assisted in the designing and planning of this project. All authors have read and approved the manuscript.
